# Artificial Intelligence‐Enhanced Navigation for Early Detection of Inferior Vena Cava and Root of the Major Hepatic Veins During Robotic Hepatectomy

**DOI:** 10.1002/jhbp.12195

**Published:** 2025-07-31

**Authors:** Yutaka Nakano, Yosuke Uematsu, Yuta Abe, Masashi Takeuchi, Minoru Kitago, Yasushi Hasegawa, Shutaro Hori, Masayuki Tanaka, Hirofumi Kawakubo, Yuko Kitagawa

**Affiliations:** ^1^ Department of Surgery Keio University School of Medicine Tokyo Japan

**Keywords:** artificial intelligence, early detection, inferior vena cava, major hepatic veins, robotic hepatectomy

## Abstract

Nakano and colleagues developed an artificial intelligence‐enhanced navigation system for robotic hepatectomy, enabling early identification of the inferior vena cava and major hepatic vein roots. Using semantic segmentation on 1030 annotated images, the model showed reliable performance and may help reduce complications, enhance safety, and support minimally invasive liver surgery.

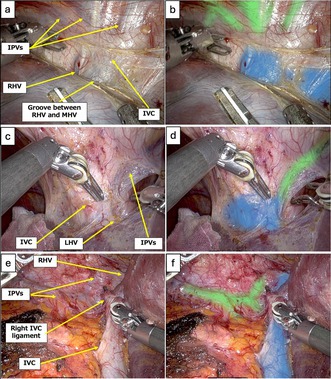

Integration of robotic surgery and artificial intelligence (AI) has the potential to revolutionize precision surgery because the robotic surgical system is equipped with a data server that allows for cloud‐based management. It can track the number of times each surgical instrument is used and records all instrument tip movements as coordinate data, making it possible to fully reproduce the operative movements performed by a particular surgeon. However, there are few studies on AI navigation in robotic hepatectomies [1]. We have focused on early detection in AI research, allowing us to avoid injury to major vessels and safely approach them as surgical landmarks. In liver surgery, injury of the inferior vena cava (IVC) and the roots of the major hepatic veins (HVs), such as the left, middle, and right hepatic veins (LHV, MHV, and RHV, respectively), occurs around the suprahepatic area. However, a safe approach to the suprahepatic IVC and root of the major HVs is important for performing the hepatic vein‐guided approach during minimally invasive anatomic liver resection, and the inferior phrenic veins (IPVs) are useful anatomical structures to safely expose the root of major HVs during minimally invasive anatomic liver resection [2].

IVC, major HVs, and IPVs were annotated as target anatomical structures by two expert surgeons in the hepatobiliary‐pancreatic field to develop an AI model focusing on the early detection of the IVC and roots of the major HVs during robotic hepatectomy. All modeling procedures were conducted using Python 3.7. Model training was performed on a workstation with an NVIDIA GeForce RTX 3090 GPU and an Intel Core i9‐10 900X CPU (3.70 GHz, 128 GB RAM). Semantic segmentation was achieved using the DeepLab v3+ architecture, which labels each pixel to identify anatomical structures. Segmentation performance was assessed by Intersection over Union (IoU), calculated as the overlap between the predicted mask and expert‐annotated ground truth divided by their union because computing the IoU across all images as a whole provides an evaluation that emphasizes the balance of the total segmented area. An IoU of 1 indicated perfect agreement, while 0 indicated no overlap. As a result, 34 cases of robotic hepatectomy were included, and 1030 images were annotated. Mean IoU was 0.487 for the IVC and major HVs, and 0.492 for the IPVs (Figure 1). Video S1 shows the AI model outputs, focusing on (i) exposing the IVC around the root of RHV and MHV, (ii) exposing the left triangular ligament and root of the LHV, and (iii) robotic right liver mobilization.

**FIGURE 1:**
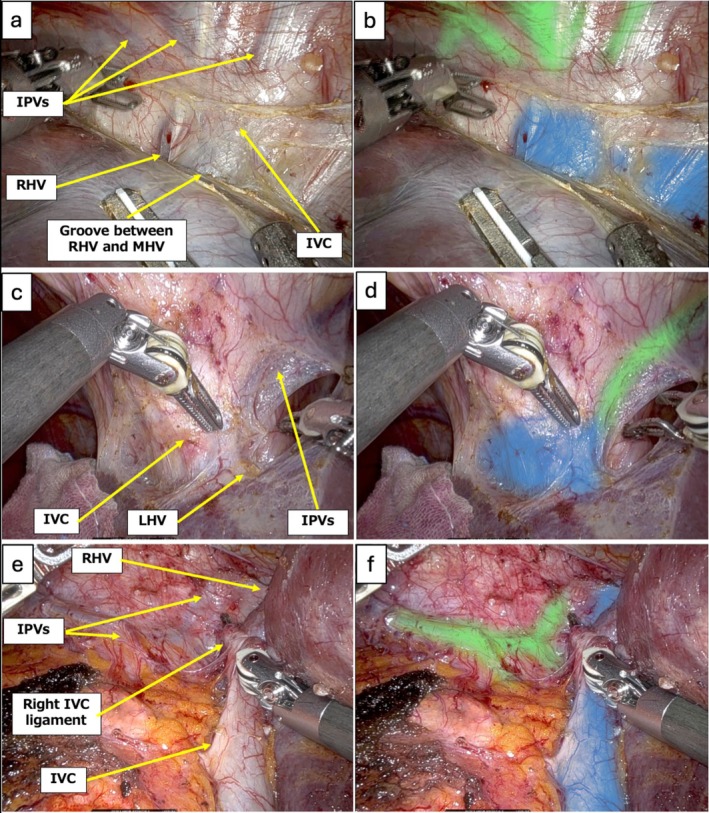
The IVC around the RHV and MHV, and the root of LHV and RHV are shown in blue, and IPVs in green, highlighted by the AI navigation system. (a, b) Exposing the IVC around the RHV and MHV without (a) and with (b) AI outputs. (c, d) The root of LHV and IPVs without (c) and with (d) AI outputs. (e, f) IVC, the root of RHV and IPVs without (e) and with (f) AI outputs during right hepatic lobe mobilization. AI, artificial intelligence; IPVs, inferior phrenic veins; IVC, inferior vena cava; LHV, left hepatic vein; MHV, middle hepatic vein; RHV, right hepatic vein.

Early detection allows us to avoid injury to the major vessels. Critical intraoperative bleeding occasionally occurs during liver surgery, particularly in the suprahepatic area. Surgical complications result from a lack of recognition and attention during surgery [3]. Therefore, to prevent vascular structure misidentification, early detection is important to reduce the adverse events and complications associated with liver surgery. However, early detection allows us to safely use them as surgical landmarks. The hepatic vein‐guided approach is a preferred technique for safely performing minimally invasive anatomic liver resection [2]. Exposure of the major HVs is the first step in this approach. Therefore, this AI model should help us perform this approach safely and smoothly for early detection of surgical landmarks. Currently, this system is being evaluated under a research protocol, which permits validation only through recorded operative images. Based on the result of this study, we aim to obtain regulatory approval as a real‐time navigation medical device in the near future.

AI navigation should be useful for the early detection of the IVC and root of major HVs, which has the potential to prevent intraoperative complications and reduce operative time. Further data accumulation to improve the AI model quality and further studies to evaluate this model for early detection are required.

## Author Contributions

Y.N. and Y.U. participated in the study conception, data acquisition, annotation, and manuscript drafting. Y.A., M.T., Y.U., and Y.K. participated in the study conception, data acquisition, and interpretation. M.K., Y.H., S.H., M.T., and H.K. participated in data acquisition. All authors approved the final version of the manuscript.

## Conflicts of Interest

Y.K. received personal fees from Intuitive Surgical G.K., personal fees from AI Medical Service Inc., grants from Medicaroid Corporation, grants and personal fees from SYSMEX CORPORATION, outside the submitted work; and The Japanese Society of Gastroenterological Surgery Editor in Chief of Annals of Gastroenterological Surgery. Y.K. has equity in Direava Inc. M.T. is the chief executive officer of Direava Inc.

## Supporting information


**Video S1:** AI model outputs demonstrating (i) IVC exposure around RHV and MHV roots, (ii) dissection of the left triangular ligament and LHV root, and (iii) robotic right liver mobilization.

## Data Availability

The data that supports the findings of this study are available in the Supporting Information of this article.
